# Serum Zinc, Copper, and Other Biometals Are Associated with COVID-19 Severity Markers

**DOI:** 10.3390/metabo11040244

**Published:** 2021-04-15

**Authors:** Anatoly V. Skalny, Peter S. Timashev, Michael Aschner, Jan Aaseth, Lyubov N. Chernova, Vladimir E. Belyaev, Andrey R. Grabeklis, Svetlana V. Notova, Ryszard Lobinski, Aristides Tsatsakis, Andrey A. Svistunov, Victor V. Fomin, Alexey A. Tinkov, Peter V. Glybochko

**Affiliations:** 1 World-Class Research Center “Digital Biodesign and Personalized Healthcare”, IM Sechenov First Moscow State Medical University (Sechenov University), 119435 Moscow, Russia; skalny3@gmail.com (A.V.S.); timashev.peter@gmail.com (P.S.T.); michael.aschner@einsteinmed.org (M.A.); jaol-aas@online.no (J.A.); whatsthegistdoctor@gmail.com (L.N.C.); belyaev_v_e@staff.sechenov.ru (V.E.B.); andrewgrabeklis@gmail.com (A.R.G.); snotova@mail.ru (S.V.N.); Ryszard.Lobinski@univ-pau.fr (R.L.); aristsatsakis@gmail.com (A.T.); svistunov@sechenov.ru (A.A.S.); fomin_v_v_1@staff.sechenov.ru (V.V.F.); rector@sechenov.ru (P.V.G.); 2 Laboratory of Medical Elementology, KG Razumovsky Moscow State University of Technologies and Management, 109004 Moscow, Russia; 3 Chemistry Department, Lomonosov Moscow State University, 119991 Moscow, Russia; 4 Department of Polymers and Composites, N.N. Semenov Institute of Chemical Physics, 119991 Moscow, Russia; 5 Department of Molecular Pharmacology, Albert Einstein College of Medicine, Bronx, NY 10461, USA; 6 Research Department, Innlandet Hospital Trust, 2380 Brumunddal, Norway; 7 Institute of Bioelementology, Orenburg State University, 460018 Orenburg, Russia; 8 l’Institut des Sciences Analytiques et de Physico-chimie pour l’Environnement et les Matériaux, Universite de Pau et des Pays de l’Adour, E2S, CNRS, 64000 Pau, France; 9 Laboratory of Toxicology, Medical School, University of Crete, Voutes, 70013 Heraklion, Crete, Greece; 10 Laboratory of Ecobiomonitoring and Quality Control, Yaroslavl State University, 150003 Yaroslavl, Russia

**Keywords:** novel coronavirus infection, lung damage, trace elements, selenium, iron

## Abstract

The objective of the present study was to evaluate of serum metal levels in COVID-19 patients with different disease severity, and to investigate the independent association between serum metal profile and markers of lung damage. The cohort of COVID-19 patients consisted of groups of subjects with mild, moderate, and severe illness, 50 examinees each. Forty-four healthy subjects of the respective age were involved in the current study as the control group. Serum metal levels were evaluated using inductively-coupled plasma mass-spectrometry. Examination of COVID-19 patients demonstrated that heart rate, respiratory rate, body temperature, C-reactive protein levels, as well as lung damage increased significantly with COVID-19 severity, whereas SpO_2_ decreased gradually. Increasing COVID-19 severity was also associated with a significant gradual decrease in serum Ca, Fe, Se, Zn levels as compared to controls, whereas serum Cu and especially Cu/Zn ratio were elevated. No significant group differences in serum Mg and Mn levels were observed. Serum Ca, Fe, Se, Zn correlated positively with SpO_2_, being inversely associated with fever, lung damage, and C-reactive protein concentrations. Opposite correlations were observed for Cu and Cu/Zn ratio. In regression models, serum Se levels were inversely associated with lung damage independently of other markers of disease severity, anthropometric, biochemical, and hemostatic parameters. Cu/Zn ratio was also considered as a significant predictor of lower SpO_2_ in adjusted regression models. Taken together, these findings demonstrated that metal metabolism significantly interferes with COVID-19 pathogenesis, although the causal relations as well as precise mechanisms are yet to be characterized.

## 1. Introduction

CoronaVirus Disease 2019 (COVID-19) is a viral infection caused by coronavirus SARS-CoV-2 which to date has already affected nearly 70 million people worldwide, resulting in more than 1.5 million deaths. The virus enters the organism through interaction with the angiotensin-converting enzyme 2 (ACE2) receptor on type II pneumocytes and predominantly affects the respiratory system causing viral pneumonia [1]. In addition, COVID-19 is characterized by pronounced systemic immunopathological effects that contribute significantly to disease pathogenesis. Briefly, lymphopenia and lymphocyte dysfunction are associated with overproduction of proinflammatory cytokines causing cytokine storm damaging organs and tissues [2]. Therefore, modulation of these pathways is considered as a therapeutic strategy in management of COVID-19 [3].

Epidemiological studies revealed a variety of physiological and environmental factors increasing the risk of COVID-19 severity. Advanced age, as well as certain morbidities, including diabetes, cardiovascular diseases, chronic kidney disease, and others are considered the major risk factors for severe COVID-19 [4]. Obesity, being the most widespread nutritional disorder, has been shown to significantly increase the risk of COVID-19 [5]. In parallel with macronutrient intake patterns, altered micronutrient status could be also considered as a potential modifiable risk factor for COVID-19 [6] especially in view of high rate of micronutrient deficiencies in COVID-19 patients [7].

Essential metals (biometals) are micronutrients involved in the organism functioning through their signaling, catalytic, cofactor, and structural role. Essential metals including Fe, Cu, Mn, Zn, and a metalloid Se are involved in regulation of immune system functioning including inflammatory response and antiviral immunity. Moreover, Zn was shown to possess direct antiviral activity through inhibition of SARS-CoV replication in vitro [8]. Based on these data, since the very beginning of COVID-19 pandemic certain metals including Zn [9], Cu [10], Se [11] were considered as the potential agents in disease management.

In parallel with findings of essential element deficiency, certain studies demonstrated the association between serum/plasma metal levels and COVID-19 severity. Specifically, dysregulation of Fe metabolism was also considered as a potential risk factor of COVID-19 severity. Specifically, serum Fe significantly correlated with COVID-19 severity both before and after treatment, as well as predicted progression from mild and moderate to severe and critical illness [12], being also associated with inflammatory cytokine levels [13]. Serum Zn levels were also found to be lower in severe COVID-19 cases and acute respiratory distress syndrome [14] as compared to mild-to-moderate illness, being considered as a significant predictor of critical illness [15]. Serum total Se and selenoprotein P levels were found to be associated with improved outcome in COVID-19 cases [16]. In turn, Ca levels were also found to be lower in critical illness, being associated with inflammatory cytokine levels and COVID-19-associated organ damage [17]. At the same time, data from comparative analysis of the association between biologically essential metals and multiple markers of COVID-19 severity are lacking.

Therefore, the objective of the present study was to evaluate of serum metal levels in COVID-19 patients with different disease severity, and to investigate the independent association between serum metal profile and markers of lung damage.

## 2. Results

The COVID-19 patients we examined had a statistically significant decrease in SpO_2_ compared to the control values of 3%, 3%, and 11% in patients with mild, moderate, and severe disease, respectively (Table 1). A reduction in arterial blood oxygen saturation was accompanied by tachypnoea, tachycardia, and fever. Specifically, in patients with mild, moderate, and severe COVID-19, the heart rate and respiratory rate exceeded the respective control values by 13% and 10%, 15% and 10%, and 19% and 23%, respectively. It is also notable that the respiratory rate in subjects with severe disease exceeded that in patients with mild and moderate COVID-19 by 12% and 11%, respectively. Maximal body temperature in the examined patients was 4% to 5% higher than in controls depending on the group. Computer tomography demonstrated that the percentage of lung damage in patients with moderate and severe COVID-19 was greater than 50% and 120% higher compared to mild disease. The studied clinical markers of disease severity and lung damage were tightly associated with systemic inflammatory response. Specifically, hs-CRP levels in patients with mild, moderate, and severe COVID-19 were more than 22-, 34-, and 82-fold higher than the respective control values. Moreover, circulating hs-CRP levels in subjects with severe COVID-10 exceeded those in the mild and moderate disease groups by a factor greater than 3.5 and 2.4, respectively.

Routine biochemical parameters indicative of systems dysfunction were also monitored in the COVID-19 patients along with markers of hemostasis (Table 2). Total protein levels in subjects with mild and moderate COVID-19 were 8% lower than in controls. The lowest serum total protein concentration was revealed in patients with severe COVID-19 were reduced by 15% and 8% as compared to healthy controls and patients with mild/moderate disease, respectively. No significant group difference in total bilirubin or creatinine levels were observed after Bonferroni adjustment. At the same time, progression of disease severity was associated with increased blood glucose levels, being the highest in subjects with severe COVID-19, exceeding the respective values in controls and patients with mild and moderate disease by 32%, 28%, and 23%, respectively. Increasing severity of COVID-19 was associated with a significant 2-fold increase in serum AST and ALT activity when compared to healthy subjects.

Markers of blood coagulation were also significantly affected by COVID-19. Particularly, INR values in patients with mild, moderate, and severe disease were 21%, 23%, and 36% higher than those in healthy subjects, respectively. Prothrombin percentage was characterized by a significant 20%, 24%, and 29% decrease as compared to the respective control values. Fibrinogen levels in subjects with mild, moderate, and severe disease were found to be 2-, 2.1-, and 2.8-fold higher than those in healthy controls, respectively. It is also notable that fibrinogen levels in a group of patients with severe disease significantly exceeded that in subjects with mild and moderate COVID-19 by 40% and 32%, respectively. No significant difference in prothrombin time and activated partial thromboplastin time (APTT) were revealed.

**Figure 1:**
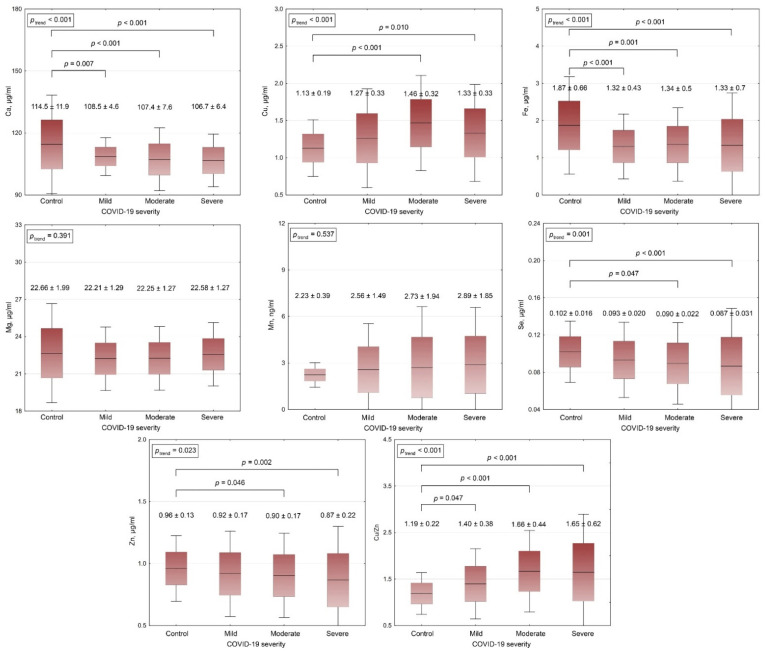
Serum metal levels and Cu-to-Zn ratio in COVID-19 patients in relation to disease severity. Data are expressed as mean ± SD; *p* values are indicated according to one-way ANOVA Bonferroni’s adjustment.

Serum ICP-MS analysis revealed significant alterations in biometal levels in patients with COVID-19 (Figure 1). Only serum Cu level was characterized by a significant increase in patients with mild, moderate, and severe COVID-19, being 12%, 29%, and 18%, respectively, higher than the respective values in healthy controls. Serum Mn concentration also trended higher in COVID-19 patients, although the differences were not significant due to high variability in this parameter. Circulating levels of other metals were reduced upon COVID-19 infection. Specifically, serum Fe levels in all groups of patients were ~29% lower than the respective values in the control group. In turn, serum Se concentrations in patients with mild, moderate, and severe COVID-19 were 9%, 12%, and 15%, respectively, lower than in healthy subjects. Circulating Zn levels also reduced significantly in association with COVID-19 severity, being 4%, 6%, and 9% lower in subjects with mild, moderate, and severe disease, respectively, compared to healthy controls. Serum Ca levels were found to be 5%, 6%, and 7% lower in subjects with mild, moderate, and severe COVID-19 when compared to the control values. No significant group difference or trend in serum Mg levels were noted between patients and controls.

In view of significant bidirectional changes of serum Cu and Zn levels, serum copper-to-zinc (Cu/Zn) ratio was also evaluated in the studied cohort. Cu/Zn values were found to increase gradually in association with COVID-19 severity. Specifically, patients with mild, moderate, and severe COVID-19 were characterized by 18%, 39%, and 39% higher Cu/Zn ratio, respectively, than in healthy controls.

**Figure 2:**
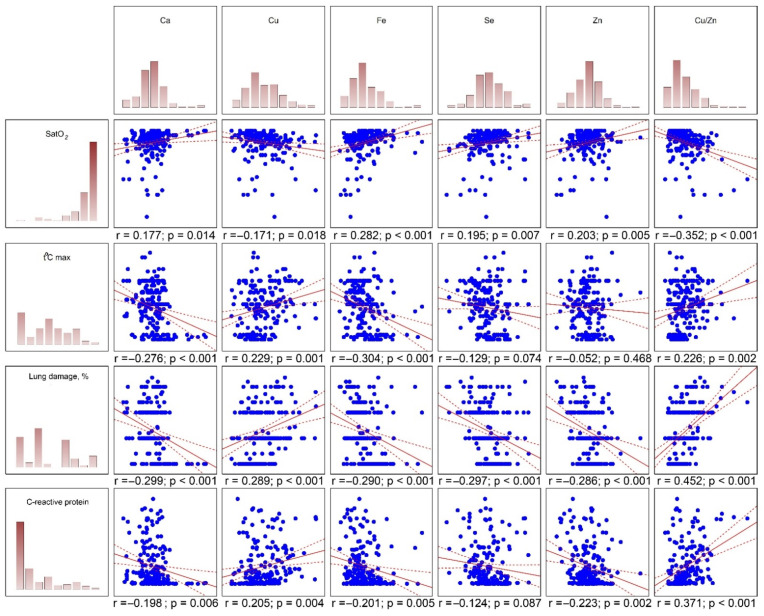
Correlation between serum metal levels and markers of disease severity.

Correlation analysis was performed to determine crude relationship between serum metal levels and markers of the disease severity (Figure 2). Serum Fe, and to a lesser extent Se, Zn, and Ca correlated directly with SpO_2_, whereas circulating Cu and especially Cu/Zn ratio were inversely associated with blood oxygen saturation. Conversely, circulating Ca, Fe, Se, and Zn levels were characterized by an inverse correlation with lung damage, CT grade, CRP levels, as well as fever. In turn, serum Cu and Cu/Zn ratio were tightly associated with markers of lung damage (relative lung damage, CT grade), inflammation (CRP), and fever (t °C_max_). In corroboration of the outcome of group comparisons, both serum Mn and Mg levels did not correlate significantly with SpO_2_, fever, lung damage, CT grade, or CRP levels.

Multiple linear regression analysis was performed in order to specify the association between serum metal levels and markers of disease severity, SpO_2_ (Table 3) and lung damage (Table 4) as dependent variables.

A crude model (Model 1) incorporating only the levels of metals (Ca, Cu, Fe, Se, Zn) and Cu/Zn ratio demonstrated a significant inverse association between serum Cu and Cu/Zn ratio with SpO_2_, whereas circulating Fe and Zn levels were considered as positive predictors. After adjustment for age, gender, BMI, HR, RR, and t °C_max_ (Model 2) serum Cu, Cu/Zn (negative), and Zn (positive) remained significantly associated with SpO_2_. Among the confounders used, age and percentage of lung damage were found to be significantly inversely associated with SpO_2_ values. Model 3 was additionally adjusted for biochemical and hemostasis markers that were significantly affected by COVID-19 infection. The revealed associations between serum Cu, Zn, and Cu/Zn with SpO_2_ remained significant. Serum Fe levels were found to be nearly significantly associated with SpO_2_ in a positive manner. In Model 3 age, hs-CRP, and serum AST activity were also considered as negative predictors of SpO_2_. The overall models 1, 2 and 3 accounted for up to 20%, 34%, and 42% of SpO_2_ variability.

Lung damage was found to be inversely associated with serum Ca and Fe levels, whereas Cu/Zn ratio appeared to be a positive predictor in a crude model (Model 1). Subsequent adjustment for clinical variables (Model 2) revealed inverse association between serum Ca and Se and lung damage, whereas BMI, HR, and t°C_max_ were characterized by a direct interrelation with the latter. At the same time, SpO_2_ was considered as the most potent negative predictor of lung damage. In Model 3 additionally adjusted for biochemical and hemostatic parameters serum Se levels remained as a significant negative predictor of lung damage, as did SpO_2_ and total protein levels. In turn, INR values were characterized by a strong positive association with lung damage. While all being significant, models 1, 2 and 3 accounted for 28%, 53% and 61% of lung damage (%) variability, respectively, in the studied cohort.

**Figure 3:**
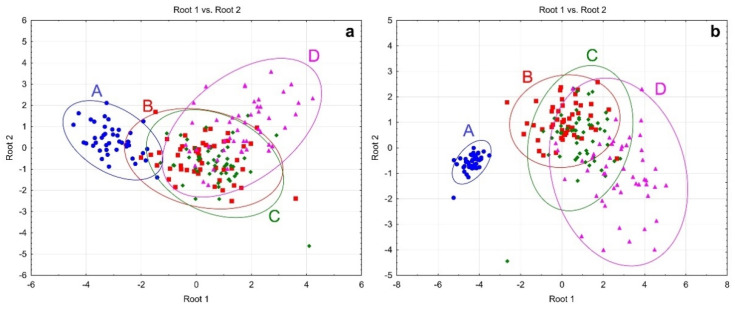
Scatter plot of canonical discriminant analysis based on serum metal levels (**a**) and clinical, biochemical, hemostatic parameters, as well as serum metal levels (**b**). A—control group (blue), B—Mild (red), C—Moderate (green), D—Severe (purple).

The contribution of the studied metals to the difference between the groups was evaluated by canonical discriminant analysis. Model 1 (Figure 3a) was based only on serum metal levels. The distance between control and cases groups centroids was significant (*p* < 0.001), although the model provided complete discrimination only between the control and severe COVID-19 group. Discriminant function analysis revealed a significant contribution of serum Ca (*p* < 0.001), Cu (*p* = 0.003), Fe (*p* = 0.002), and Cu/Zn (*p* = 0.002) values into the model.

In order to reveal contribution of the studied metals into integral differences between cases and controls, Model 2 was adjusted for anthropometric and clinical variables, as well as biochemical and hemostatic markers (Figure 3b). Despite significant distances between the group centroids, the current model resulted only in a complete discrimination for the control group from the COVID-19 patients. At the same time, complete discrimination between groups of patients with mild, moderate, and severe COVID-19 was not observed based on the current model. Discriminant function analysis demonstrated that of all parameters in the model SpO_2_, t°C_max_, CRP, INR, PT, lung damage (all *p* < 0.001), fibrinogen levels (*p* = 0.007), as well as Cu (*p* = 0.002) and Cu/Zn (*p* < 0.001) were characterized by a significant contribution to group discrimination.

## 3. Discussion

The results of the present study demonstrate that COVID-19 is associated with alteration serum biometal profile, characterized by decreased circulating Ca, Fe, Se, and Zn levels and elevation of Cu and Cu/Zn values. Serum metal levels were also significantly associated with COVID-19 severity after adjustment for anthropometric, clinical, and laboratory variables, indicative that metal levels are altered in a disease severity-dependent manner. These findings generally highlight the potential involvement of metal metabolism in COVID-19 pathology.

We report that serum Se levels were lower in COVID-19 patients, and inversely associated with lung damage. Indirect data demonstrated that population-wide Se status is associated with higher COVID-19 cure rate in regions with higher hair Se content [18]. Evaluation of serum Se levels revealed 13% lower values in Indian COVID-19 patients compared to healthy controls [19]. Correspondingly, both serum Se and selenoprotein P (SELENOP) levels were found to be lower in COVID-19 patients with low values (<2.5 of the reference population) observed in 43.4% and 39.2% cases, respectively. Moreover, lower serum Se and SELENOP levels were found to be associated with higher COVID-19 mortality [14]. Further analysis demonstrated that combined use of serum SELENOP and Zn along with age might be considered a reliable predictor of COVID-19 survival [20]. These observations corroborate observations on the involvement of Se and selenoprotein metabolism in COVID-19 pathogenesis which is known to be mediated by modulation of redox homeostasis and endoplasmic reticulum stress, with subsequent regulation of inflammatory and immune signaling [21], as well as viral replication [22]. Current understanding of the role of host Se status as a significant determinant of viral infection provided a rationale for recommendations of Se supplementation for reduction of COVID-19 severity, especially in low-Se populations [11].

Serum Fe levels were found to be lower in COVID-19 patients, and associated with improved blood oxygen saturation and lower disease severity markers. Serum Fe deficiency was also shown to be associated with COVID-19 severity and valuable in predicting transition from mild to severe illness, as well as related to higher patient mortality [12]. In addition, patients with persistent alterations in Fe metabolism were characterized by higher rate of lung damage and reduced performance [15]. These findings are generally in agreement with earlier observation on low transferrin saturation in COVID-19 patients admitted to intensive care units [23]. Correspondingly, an increase in ferritin/transferrin ratio was associated with a substantially elevated risk of ICU admission and mechanical ventilation [13]. The observed decrease in serum Fe in COVID-19 patients may be mediated by overproduction of proinflammatory cytokines and subsequent up-regulation of hepcidin production, corroborating the high rate of anemia of inflammation in COVID-19 patients [13]. Correspondingly, increased serum hepcidin levels were found to be a significant predictor of COVID-19 severity independent of circulating ferritin concentrations [24]. The observed positive relationship between serum iron and blood oxygen saturation is attributed to the role of Fe in oxygen transport, corroborating earlier findings of inverse association between serum Fe and hypoxemia severity [25].

The noted gradual decrease in serum Zn levels and its association with markers of disease severity are consistent with potential association between Zn metabolism and COVID-19 [9]. Earlier studies provided evidence on the association between Zn deficiency and COVID-19. Specifically, Zn-deficient patients were characterized by higher rate of COVID-19 complications and longer hospital stays that may be at least partially mediated by the modulatory effect of Zn on SARS-CoV-2 spike protein interaction with angiotensin-converting enzyme 2 (ACE2) [26]. Lower Zn levels were also observed in SARS-CoV-2 positive pregnant women [27]. In view of the earlier demonstrated in vitro Zn-dependent inhibition of SARS-CoV viral replication [8], as well as immunomodulatory, antioxidant, anti-inflammatory role of the metal, its use in management of COVID-19 has been focus of intense interest [9,28]. However, experimental data from only single trials are available to date, and they are contradictory. In particular, administration of zinc sulfate along with hydroxychloroquine and azithromycin significantly improved clinical outcome, reduced risk of mechanical ventilation, and mortality when compared to the protocol lacking Zn supplementation [29]. At the same time, another trial failed to reveal any significant beneficial effect of Zn supplementation on COVID-19 outcome [30]. It has been proposed that the difference in baseline Zn status might underlie differential effects of Zn supplementation in COVID-19 patients. Therefore, further trials are required to estimate the efficiency of Zn in management of COVID-19 [31], although in general, Zn supplementation appears to be of clinical efficacy [9].

Low serum Ca levels were found to be associated with markers of lung damage and disease severity in COVID-19. Previous studies have also revealed significantly reduced serum total as well as ionized Ca levels in COVID-19 patients [32]. Serum Ca levels were found to correlate inversely with inflammation as assessed by white blood cell count, IL-6, and CRP levels, as well as D-dimer and procalcitonin levels [33], as well as organ injury in COVID-19 [26]. Hypocalcemia was shown to be frequent in COVID-19 patients, being associated with higher risk of ICU admission and mortality [34], as well as higher requirement in high oxygen support [35]. In agreement, low serum Ca levels were found to be more prevalent in severe and critical COVID-19 cases than in moderate illness [36]. It is proposed that vitamin D deficiency may at least partially underlie the observed alterations in Ca metabolism in COVID-19 [37].

In contrast to other metals studied, only serum Cu levels were characterized by a significant gradual increase in association with disease severity. These findings are in agreement with a recent study demonstrating 16% lower serum Cu levels in Indian COVID-19 infected pregnant women when compared to non-infected counterparts [27]. In contrast, an earlier study proposed the use of copper as adjunct therapy for COVID-19 management due to its potential antiviral effects, although the authors also mention that the use of copper may be limited by its toxicity [10]. The present study demonstrated an association between serum Cu levels and COVID-19 severity including circulating hs-CRP level, in agreement with the proinflammatory activity of the metal [38].

In parallel to the elevation of serum Cu and decrease in Zn levels, COVID-19 patients exhibited a more profound increase in serum Cu/Zn ratio. Moreover, Cu/Zn values were found to be more tightly associated with markers of disease severity than Cu and Zn solely, being indicative of the potential interactive (antagonistic) effects between these biometals that may be attributed to differential effects on redox homeostasis and inflammatory pathways [39]. Correspondingly, Cu/Zn ratio was considered as the potential biomarker of inflammation and all-cause mortality in elderly population [40]. In addition, elevated Cu/Zn ratio was shown to be associated with higher risk of infectious diseases leading to hospitalization [41].

## 4. Materials and Methods

The protocol of investigation was evaluated and approved by the Institutional Ethics Committee of Sechenov University (Moscow, Russia). All procedures performed within the study were in agreement with the principles of the Declaration of Helsinki (1964) and its later amendments (Ethical approval code: 07-17/13.09.17).

This prospective observational study was performed between March and June 2020, involving 150 patients with COVID-19 who admitted Departments for treatment of patients with COVID-19 of the Sechenov University Clinical center. Diagnosis was verified by RT-PCR testing for SARS-CoV-2-positivity. Only patients who admitted the inpatient department were involved in the current study.

The cohort of COVID-19 patients consisted of groups of subjects with mild, moderate, and severe illness, 50 examinees each. Classification of COVID-19 severity was performed using the guidelines of the Ministry of Healthcare: mild—fever < 38 °C, cough, weakness, lack of criteria of moderate and severe illness; moderate—fever > 38 °C, HR > 22, dyspnea in response to physical activity, pneumonia verified using computer tomography (CT stages 1-2 of lung damage), SpO_2_ < 95%, CRP > 10 mg/L; severe—HR > 30, SpO_2_ < 93%, PaO_2_/FiO_2_ < 300 mmHg, lung damage progression revealed using CT, appearance of signs of other diseases, altered consciousness, unstable hemodynamics (systolic arterial pressure < 90 mmHg or diastolic arterial pressure < 60 mmHg, diuresis < 20 mL/h), arterial blood lactate > 2 mmol/L, qSOFA > 2; acute respiratory insufficiency and requirement in mechanical ventilation, septic shock, multiple organ failure [42]. Forty-four healthy subjects of the respective age were involved in the current study as the control group (Table 5). In view of high variability in the anthropometric data, all statistical analyses were adjusted for age, gender, and BMI values.

Body weight (kg) and height (m) were recorded with subsequent calculation of body mass index (BMI) according to a standard formula. Physical examination also Ied registration of respiratory rate (RR), heart rate (HR), as well as body temperature with the use of maximal values for statistical analysis (t °C_max_). Blood oxygen saturation was evaluated using pulse oximeter. Computer tomography was used for evaluation of lung damage as percentage (%) of total lung tissue with subsequent grading of lung damage into four stages according to national guidelines [18].

Blood samples were taken from the cubital vein into separate tubes containing sodium citrate for analysis of hemostasis parameters, as well anticoagulant-free tube for analysis of routine biochemical parameters and metal levels. Serum/plasma samples were obtained using centrifugation at 1600× *g* for 10 min.

Evaluation of serum total protein, total bilirubin, creatinine, glucose, alanine aminotransferase (ALT), aspartate aminotransferase (AST), and C-reactive protein levels was performed using the respective Randox kits (Randox Laboratories Ltd., Crumlin, UK) at an automated biochemical analyzer. Plasma markers of hemostasis Iing fibrinogen, prothrombin, activated partial thromboplastin time (APTT), and prothrombin time (PT) were evaluated on a MaxMat PL Coag analyzer (Maxmat SA, Montpellier, France).

For serum metal analysis the obtained serum samples were diluted 1:15 (*v*/*v*) with an acidified diluent (pH = 2.0) consisting of 1-butanol 1% (Merck KGaA, Darmstadt, Germany), Triton X-100 0.1% (Sigma-Aldrich, Co., St. Louis, MO USA), and HNO_3_ 0.07% (Sigma-Aldrich, Co.) in 18.2 MΩ · cm distilled deionized water (Labconco Corp., Kansas City, MO, USA). Evaluation of calcium (Ca), copper (Cu), iron (Fe), magnesium (Mg), manganese (Mn), selenium (Se), and zinc (Zn) levels in serum samples was performed using inductively-coupled plasma mass-spectrometry (ICP-MS) at NexION 300D spectrometer (Perkin Elmer Inc., Shelton, CT, USA) equipped with 7-port FAST valve and ESI SC-2 DX4 autosampler (Elemental Scientific Inc., Omaha, NE, USA). Calibration of the ICP-MS system was performed with 0.5, 5, 10 and 50 μg/L solutions of the studied elements prepared from the commercially available Universal Data Acquisition Standards Kit (Perkin Elmer Inc.). The solutions (10 μg/L) of yttrium-89 and rhodium-103 prepared from Yttrium (Y) and Rhodium (Rh) Pure Single-Element Standard (Perkin Elmer Inc.) were used for internal online standardization. The obtained data on serum metal levels were expressed as µg/mL for all metals except Mn (ng/mL). Laboratory quality control was performed daily using the certified reference materials of human plasma (ClinChek^®^ Plasma Control, Levels I, II, Lot 1286, RECIPE Chemicals + Instruments GmbH, Munich, Germany) yielding the recovery rates of 89–112% for all metals assessed.

Statistical treatment of the obtained data was performed using Statistica 10 (Statsoft, Tulsa, OK, USA). The obtained data are expressed as mean and the respective standard deviation (Mean ± SD). Data distribution was assessed using Shapiro-Wilk test with subsequent log-transformation of the raw data characterized by skewed distribution. Group comparisons were performed using one-way analysis of variance (ANOVA) with Bonferroni adjustment. Spearman’s rank coefficient was used for correlation analysis. Multiple linear regression was performed in order to reveal the contribution of serum metal levels (independent variables) to variability of COVID-19 severity markers including SpO_2_ and lung damage (dependent variables) after adjustment for confounding anthropometric, biochemical, and hemostatic parameters. Model 1 (crude) incorporates only serum metal levels, Model 2—adjusted for anthropometric and clinical data, Model 3—adjusted for routine biochemical and hemostatic parameters. In addition, canonical discriminant analysis was performed in order to reveal the potential contribution of serum metal levels into discrimination between the groups with evaluation of significance of the distance between group vectors. The level of significance was set at *p* < 0.05 for all statistical analyses.

## 5. Conclusions

The results of the present study demonstrated significant alterations in biometal levels in patients with COVID-19, as well as associations between biometal levels and disease severity. Particularly, COVID-19 was associated with a significant reduction in serum Ca, Fe, Se, and Zn levels, which inversely correlated with fever, lung damage, and inflammation, and positively correlated with SpO_2_. These findings support the premise that essential metal deficiency represents as a risk factor for COVID-19. In contrast, serum Cu levels and Cu/Zn ratio were increased in COVID-19 cases, and tightly related to markers of disease severity. Multivariate analysis demonstrated that altered metal levels were associated with COVID-19 independently of anthropometric, biochemical, and hemostatic markers. Multiple linear regression demonstrated that serum Se levels represent an independent predictor of lung damage, whereas serum Cu and Cu/Zn ratio were independent determinants of reduced SpO_2_. Taken together, our novel findings demonstrated that metal metabolism significantly interferes with COVID-19 pathogenesis, although the causal relations as well as precise mechanisms have yet to be characterized. Investigation of the association between metal metabolism and the balance between anti- and proinflammatory cytokine production could provide data on the potential mechanisms of involvement of metals into COVID-19 pathogenesis. Further studies are required to clarify the particular pathways affected by COVID-19-associated alterations in metal metabolism, as well as to estimate the potential benefits of essential metal supplementation in the management of COVID-19.

## Figures and Tables

**Table 1:** Characteristics of the disease severity in COVID-19 patients.

Parameter	Control	Mild	Moderate	Severe	Trend *p*
SatO_2_, %	97.98 ± 0.83	95.43 ± 1.81	94.76 ± 2.15 ^1^	86.98 ± 8.55 ^1,2,3^	<0.001
HR, per min	73.23 ± 6.22	83.02 ± 14.94 ^1^	84.43 ± 10.64 ^1^	86.94 ± 13.23 ^1^	<0.001
RR, per min	18 ± 1.54	19.74 ± 2.22 ^1^	19.94 ± 2.29 ^1^	22.15 ± 4.69 ^1,2,3^	<0.001
Fever, t °C_max_	36.6 ± 0.09	38.21 ± 0.68 ^1^	38.12 ± 0.63 ^1^	38.32 ± 0.8 ^1^	<0.001
Lung damage, %	-	26.22 ± 10.33	39.7 ± 16.15 ^2^	57.9 ± 17.11 ^2,3^	<0.001
CRP, mg/L	1.95 ± 1.29	44.57 ± 65.28 ^1^	67 ± 66.34 ^1,2^	161.28 ± 88.61 ^1,2,3^	<0.001

Data expressed as mean ± SD (continuous variables) and *n* (%) for categorical variables (CT grade); HR—heart rate, RR—respiratory rate, CT—computer tomography, CRP—C-reactive protein; ^1,2,3^ —significant group difference as compared to groups 1 (Control), 2 (mild illness), and 3 (moderate illness) according to one-way ANOVA with Bonferroni adjustment, respectively.

**Table 2:** Biochemical and hemostatic variables in patients with different COVID-19 severity in comparison to healthy controls.

Parameter	Control	Mild	Moderate	Severe	Trend *p*
Total protein, g/L	77.61 ± 3.87	71.66 ± 6.36 ^1^	71.72 ± 7.2 ^1^	66.21 ± 6.76 ^1,2,3^	<0.001
Total bilirubin, µmol/L	13.27 ± 6.46	10.15 ± 4.11	10.6 ± 6.81	10.4 ± 5.05	0.074
Creatinine, µmol/L	86.31 ± 10.18	99.57 ± 34.53	92.95 ± 20.30	99.11 ± 39.03	0.534
Glucose, mmol/L	5.4 ± 0.58	5.6 ± 1.06	5.79 ± 1.62	7.15 ± 3.48 ^1,2,3^	<0.001
AST, U/L	25.44 ± 7.37	39.69 ± 27.98 ^1^	44.1 ± 38.96 ^1^	51.54 ± 30.43 ^1,2^	<0.001
ALT, U/L	23.23 ± 9.12	47.56 ± 51.39 ^1^	50.98 ± 61.56 ^1^	54.34 ± 63.65 ^1^	<0.001
INR	0.97 ± 0.08	1.17 ± 0.09 ^1^	1.19 ± 0.09 ^1^	1.32 ± 0.23 ^1,2,3^	<0.001
PT, s	13.93 ± 1.06	13.06 ± 1.63	14.22 ± 8.18	14.37 ± 2.53	0.078
Prothrombin, %	101.02 ± 6.12	80.83 ± 8.08 ^1^	76.71 ± 12.43 ^1^	71.49 ± 14.83 ^1,2^	<0.001
APTT, ratio	1.02 ± 0.11	1.04 ± 0.14	1.06 ± 0.13	1.05 ± 0.2	0.722
Fibrinogen, g/L	2.67 ± 0.64	5.32 ± 2.15 ^1^	5.62 ± 1.86 ^1^	7.44 ± 2.9 ^1,2,3^	<0.001

Data expressed as mean ± SD; AST—aspartate aminotransferase, ALT—alanine aminotransferase; INR—international normalized ratio, PT— prothrombin time, APTT— activated partial thromboplastin time; ^1,2,3^—significant group difference as compared to groups 1 (Control), 2 (mild illness), and 3 (moderate illness) according to one-way ANOVA with Bonferroni adjustment, respectively.

**Table 3:** Multiple regression analysis of the association between blood oxygen saturation (SpO_2_) (dependent variable) and serum metal levels (Model 1) after adjustment for anthropometric and clinical variables (Model 2), and biochemical and hemostatic markers (Model 3).

Parameter	Model 1		Model 2		Model 3	
	β	*p*	β	*p*	β	*p*
Ca	0.110	0.090	0.017	0.765	0.014	0.789
Cu	−0.911	<0.001 *	−0.832	<0.001 *	−0.602	0.001 *
Fe	0.179	0.009 *	0.081	0.177	0.103	0.068
Se	0.022	0.772	−0.078	0.235	−0.037	0.548
Zn	0.726	<0.001 *	0.707	<0.001 *	0.533	<0.001 *
Cu/Zn	−1.378	<0.001 *	−1.120	<0.001 *	−0.789	<0.001 *
Age	-	-	0.005	0.930	0.043	0.427
Gender	-	-	−0.330	<0.001 *	−0.269	<0.001 *
BMI	-	-	0.038	0.504	0.103	0.055
HR	-	-	−0.027	0.664	−0.004	0.945
RR	-	-	−0.055	0.380	0.012	0.838
t °C_max_	-	-	−0.099	0.145	−0.030	0.657
Lung damage	-	-	−0.343	<0.001 *	−0.223	0.006 *
CRP	-	-	-	-	−0.374	<0.001 *
Total protein	-	-	-	-	−0.128	0.053
Glucose	-	-	-	-	−0.069	0.212
AST	-	-	-	-	−0.124	0.179
ALT	-	-	-	-	0.123	0.165
INR	-	-	-	-	−0.114	0.133
PT	-	-	-	-	0.048	0.379
Fibrinogen	-	-	-	-	0.039	0.611
Multiple R	0.505		0.706		0.771	
Multiple R^2^	0.255		0.498		0.594	
Adjusted R^2^	0.231		0.462		0.544	
*p* for a model	<0.001 *		<0.001 *		<0.001 *	

Data are expressed as regression coefficient (β) and the respective *p* values; *—association is significant at *p* < 0.05; BMI—body mass index; HR—heart rate; RR—respiratory rate; CRP—C-reactive protein; AST—aspartate aminotransferase; ALT—alanine aminotransferase; INR - international normalized ratio; PT—prothrombin time.

**Table 4:** Multiple regression analysis of the association between lung damage (dependent variable) and serum metal levels (Model 1) after adjustment for anthropometric and clinical variables (Model 2), and biochemical and hemostatic markers (Model 3).

−	Model 1		Model 2		Model 3	
	β	*p*	β	*p*	β	*p*
Ca	−0.242	<0.001 *	−0.116	0.029 *	−0.082	0.098
Cu	−0.143	0.485	0.096	0.597	0.158	0.360
Fe	−0.125	0.060	0.025	0.661	0.039	0.455
Se	−0.114	0.125	−0.117	0.057	−0.121	0.033 *
Zn	0.160	0.352	−0.115	0.450	−0.123	0.385
Cu/Zn	0.561	0.026 *	0.040	0.860	−0.042	0.844
Age	-	-	−0.036	0.498	−0.073	0.151
Gender	-	-	−0.036	0.540	−0.112	0.056
BMI	-	-	0.112	0.033 *	0.058	0.247
HR	-	-	0.071	0.229	0.086	0.119
RR	-	-	0.137	0.019 *	0.083	0.130
t °C_max_	-	-	0.251	<0.001 *	0.100	0.112
SpO_2_	-	-	−0.302	<0.001 *	−0.193	0.006 *
CRP	-	-	-	-	0.080	0.344
Total protein	-	-	-	-	−0.174	0.004 *
Glucose	-	-	-	-	0.064	0.214
AST	-	-	-	-	−0.090	0.295
ALT	-	-	-	-	0.138	0.092
INR	-	-	-	-	0.231	0.001 *
PT	-	-	-	-	−0.012	0.806
Fibrinogen	-	-	-	-	0.031	0.671
Multiple R	0.553		0.747		0.805	
Multiple R^2^	0.306		0.559		0.648	
Adjusted R^2^	0.284		0.527		0.605	
*p* for a model	<0.001 *		<0.001 *		<0.001 *	

Data are expressed as regression coefficient (β) and the respective *p* values; *—association is significant at *p* < 0.05; BMI—body mass index; HR—heart rate; RR—respiratory rate; CRP—C-reactive protein; AST—aspartate aminotransferase; ALT—alanine aminotransferase; INR—international normalized ratio; PT—prothrombin time.

**Table 5:** Anthropometric variables of the studied groups.

Parameter	Control	Mild	Moderate	Severe
Age, y.o.	55.67 ± 4.36	50.47 ± 15.91	54.22 ± 12.5	64.5 ± 15.49
Height, cm	1.72 ± 0.07	168.64 ± 8.75	171.12 ± 11.63	170.19 ± 8.54
Weight, kg	78.09 ± 9.01	85.52 ± 23.08	93.88 ± 19.5	88.82 ± 16.09
BMI	26.24 ± 2.29	30.06 ± 8.35	32.09 ± 5.96	30.31 ± 5.4
Gender, m/f	27/16 (63%/37%)	25/25 (50%/50%)	31/19 (62%/38%)	25/25 (50%/50%)

Data expressed as mean ± SD (continuous variables) and *n* (%) for categorical variables (gender); BMI—body mass index.

## Data Availability

The data presented in this study are available in article.
